# A new voice for health equity in the Americas

**DOI:** 10.1016/j.lana.2021.100058

**Published:** 2021-09-07

As we approach the final months of 2021, it becomes clear that the second year of the COVID-19 pandemic will not be the last one. Since the beginning, SARS-CoV-2 has affected the various regions and populations differently, with a devastating impact in low-income and middle-income countries that will take decades to recover. But COVID-19 has also exacerbated social inequalities, unmasked rooted structural racism, and exposed broken health-care systems in higher-income countries.

Latin American countries and the Caribbean (LAC) can be included in the group hit hard by COVID-19. These countries account for 12 of every 100 new infections worldwide, and they have reported over 1 399 000 deaths so far. While countries in North America experience a resurgence of COVID-19 after a brief period of remission, LAC have never left the first wave of the pandemic. Still, if and when LAC finally have COVID-19 under control, the secondary effects of the pandemic—including deepening of social inequalities, increased poverty, and economic crises—will keep haunting these countries for a while.

It is an eminent fact by now that a pivotal tool for COVID-19 control is global immunisation, an accomplishment that would require fair and equal distribution of vaccines among all countries. This is still far from the reality. Most LAC suffer from the restricted availability of immunisation, and vaccination coverage can be as low as 2% in some Caribbean countries. Not far from the Caribbean, the USA faces a different obstacle that hampers the country from achieving a desirable vaccination coverage: vaccine hesitancy. Despite the availability of vaccines, a substantial number of Americans have chosen not to get vaccinated; a scenario mostly influenced by the dissemination of fake news and anti-science beliefs, but one that is also a result of years of marginalisation of minorities that are now reluctant to engage with public health programmes. Notably, vaccine hesitancy is not just a local phenomenon but a global one, having also affected many places in South America and, in countries such as Brazil and Mexico, it has also become a political weapon.

The pandemic, to this end, is a magnifying lens, exposing a broad range of regional issues faced by the different countries within the American continents. To help meet the health and clinical challenges in the region, the *Lancet* Group has launched a new open-access journal, *The Lancet Regional Health – Americas,* as part of *The Lancet*'s global initiative to advocate for health-care quality and access in all six WHO regions of the world. Our journal aims to foster the advance of clinical practice and health policy in the Americas through the dissemination of high-quality research with the mission of improving health outcomes. Since submissions opened in May 2021, hawse have received research articles, commentaries, letters, reviews, and health policy papers from many countries in the region, including Argentina, Belize, Brazil, Canada, Chile, Colombia, French Guiana, Peru, and the USA. In this inaugural issue, we present a selection of papers highlighting reliable, science-based evidence on health issues related to COVID-19, as well as other issues pertaining to the region.

The emergence of new SARS-CoV-2 variants of concern changed the pandemic dynamics in 2021. Two population studies in this inaugural issue explored the emergence of the gamma (P.1) variant in the state of Amazonas, Brazil. Freitas and colleagues observed changes in the pattern of mortality due to COVID-19 between age groups and gender, affecting young adults and women at a higher rate, simultaneously with the emergence of the gamma strain; while Hitchings and colleagues evaluated the effectiveness of CoronaVac vaccine against symptomatic SARS-CoV-2 infection in a cohort of health-care workers. In two commentaries, the epidemiological switch of the pandemic—called the "COVID-19 youthening phenomenon"—is discussed by Guimarães and colleagues, and the challenges of vaccine production and distribution in Latin America are discussed by Homma and colleagues. Iuliano and collaborators explored the underreporting of COVID-19 deaths in the USA, highlighting that the burden of the pandemic in the country might be bigger than previously reported. In “Unintended trauma”, Statz and Heidbrink call attention to the psychological burden inflicted to children retained in detention centres in the USA–Mexico border since the pass of the Title 42 programme in the beginning of the pandemic. Meanwhile, Groves and colleagues reported a decrease in diagnostics of all seasonal respiratory viruses during 2020–21 in Canada, a possible secondary effect of the public health measures implemented to contain the COVID-19 pandemic.

In addition to COVID-19, most LAC continue to suffer from several other endemic infectious diseases, including dengue, malaria, Chagas disease, and leishmaniosis, just to name a few. Addressing these issues requires nationwide public health initiatives and strong leadership. In a retrospective analysis, Mejia-Oquendo and colleagues reported the positive impacts of the implementation of an evidence-based guideline to prevent congenital toxoplasmosis in a high-incidence area in Colombia. Improving neonatal health is also the focus of the research by Carrilo-Larco and colleagues, who evaluated birthweight registries in all regions of Peru and identified the higher incidence of low birthweight in the highlands and regions with the worst socioeconomic indicators. Socioeconomical inequalities permeate various health issues in LAC, as well as the poor availability of epidemiological data regarding most diseases. For instance, despite the known high incidence of chronic kidney disease in the region, Lin and colleagues conducted the first nationwide study on the prevalence and risk factors of chronic kidney disease in a Caribbean country. The reported findings showed an even higher incidence of this disease in Belize than in other countries, which was associated with low awareness of the disease in the population.

The mission of *The Lancet Regional Health - Americas* is to give a voice to research scientists, clinicians, policy makers, and other relevant stakeholders by creating an open forum for constructive discussions that can help advance research and health care in the region. We are committed to advocate for universal, high-quality, and accessible health care in the region. Through connecting the multiple health and policy sectors, we hope to create a strong platform to raise awareness and stimulate public debate about regional health issues, with the ultimate goal of improving health outcomes and reducing health inequality in the Americas.

*The Lancet Regional Health - Americas*

